# Neuromuscular training to enhance sensorimotor and functional deficits in subjects with chronic ankle instability: A systematic review and best evidence synthesis

**DOI:** 10.1186/1758-2555-3-19

**Published:** 2011-09-22

**Authors:** Jeremiah O'Driscoll, Eamonn Delahunt

**Affiliations:** 1 Mount Carmel Hospital, Dublin, Ireland; 2 School of Public Health, Physiotherapy and Population Science, University College Dublin, Dublin, Ireland; 3 Institute for Sport and Health, University College Dublin, Dublin, Ireland

**Keywords:** ankle sprain, ankle instability, ankle injury, rehabilitation, injury prevention

## Abstract

**Objective:**

To summarise the available evidence for the efficacy of neuromuscular training in enhancing sensorimotor and functional deficits in subjects with chronic ankle instability (CAI).

**Design:**

Systematic review with best evidence synthesis.

**Data Sources:**

An electronic search was conducted through December 2009, limited to studies published in the English language, using the Pubmed, CINAHL, Embase, and SPORTDiscus databases. Reference screening of all included articles was also undertaken.

**Methods:**

Studies were selected if the design was a RCT, quasi RCT, or a CCT; the patients were adolescents or adults with confirmed CAI; and one of the treatment options consisted of a neuromuscular training programme. The primary investigator independently assessed the risk of study bias and extracted relevant data. Due to clinical heterogeneity, data was analysed using a best-evidence synthesis.

**Results:**

Fourteen studies were included in the review. Meta-analysis with statistical pooling of data was not possible, as the studies were considered too heterogeneous. Instead a best evidence synthesis was undertaken. There is limited to moderate evidence to support improvements in dynamic postural stability, and patient perceived functional stability through neuromuscular training in subjects with CAI. There is limited evidence of effectiveness for neuromuscular training for improving static postural stability, active and passive joint position sense (JPS), isometric strength, muscle onset latencies, shank/rearfoot coupling, and a reduction in injury recurrence rates. There is limited evidence of no effectiveness for improvements in muscle fatigue following neuromuscular intervention.

**Conclusion:**

There is limited to moderate evidence of effectiveness in favour of neuromuscular training for various measures of static and dynamic postural stability, active and passive JPS, isometric strength, muscle onset latencies, shank/rearfoot coupling and injury recurrence rates. Strong evidence of effectiveness was lacking for all outcome measures. All but one of the studies included in the review were deemed to have a high risk of bias, and most studies were lacking sufficient power. Therefore, in future we recommend conducting higher quality RCTs using appropriate outcomes to assess for the effectiveness of neuromuscular training in overcoming sensorimotor deficits in subjects with CAI.

## Introduction

The ankle joint is the second most common injured body site in sport with lateral ankle sprains being the most common type of ankle injury [1]. Thus, ankle sprains are one of the most frequently encountered musculoskeletal injuries. Ankle sprains, account for between 3% and 5% of all Emergency Department attendances in the UK, with about 5,600 incidences per day [2]. It is probable that many more attend primary care facilities, such as General Practitioners and sports clinics, and thus the true incidence may well be underestimated. In the acute phase, ankle sprains are associated with pain and loss of function, and one quarter of all injured people are unable to attend school or work for more than seven days [3].

Unfortunately, the current misconception is that ankle sprains are simple innocuous injuries. This misconception is ill placed and up to 30% of people who incur a "simple" ankle sprain will report persistent symptoms such as pain, swelling, decreased function, feelings of ankle joint instability and recurrent sprains. The generic term for these persistent symptoms is chronic ankle instability (CAI).

CAI has recently been defined as an encompassing term used to classify a subject with both mechanical and functional instability of the ankle joint [4]. Furthermore according to the definition put forth by Delahunt et al [4], to be classified as having CAI, residual symptoms such as episodes of ankle joint ''giving way'' and feelings of ankle joint instability should be present for a minimum of 1 year post-initial sprain. Mechanical instability (MI) of the ankle joint is characterized by excessive inversion laxity of the rear foot or excessive anterior laxity of the talocrural joint. As a result, joint range of motion is beyond the normal expected physiological or accessory range of motion for that joint [4]. Functional instability (FI) of the ankle joint refers to a situation whereby a subject reports experiencing frequent episodes of ankle joint ''giving way'' and feelings of ankle joint instability [4].

The well accepted paradigm put forth by Hertel [5] suggests that the development of CAI is dependent upon the interaction of various mechanical and sensorimotor insufficiencies. Mechanical insufficiencies include excessive joint laxity, restricted accessory joint gliding and micro-subluxations. Sensorimotor insufficiencies include alterations in muscle activation patterns, impaired postural stability, and altered movement patterns during gait and other functional activities.

The high rate of ankle sprains sustained during activities of daily living, occupational endeavour and across all sports, as well as the severity and subsequent negative consequences associated with the development of CAI motivates attention for preventive measures against this type of injury. Exercises to improve neuromuscular control in subjects with CAI are advocated throughout the literature [6-10], yet there remains little unequivocal evidence regarding their effectiveness. Therefore, the primary aim of this systematic review was to assess the efficacy of neuromuscular training in enhancing sensorimotor function in subjects with CAI.

## Methodology

### Literature Search

The literature search was conducted in two stages. For stage one, an initial electronic search was performed and studies were evaluated for inclusion. Stage two consisted of a hand search of the reference lists of the articles selected in stage one. The electronic search using pre-defined search terms was restricted to English-language publications found in the following databases through December 2009: PubMed (National Library of Medicine, Bethesda, MD), Embase, CINAHL, and SPORTDiscus. The latter two databases were searched simultaneously using EBSCOhost (EBSCO Industries, Inc, Birmingham, AL). The reference lists of all included articles were then checked for additional pertinent studies. The primary investigator (PI) conducted the search (*see *additional file 1)

### Article Inclusion and Exclusion Criteria

Once the search had been completed, titles and abstracts of the retrieved articles were reviewed by the PI. For final inclusion the articles had to fulfil all of the following criteria:

1) study design had to be either a randomized controlled trial (RCT), a quasi RCT, or a clinical controlled trial (CCT).

2) one of the treatment options had to consist of a neuromuscular training programme (e.g. postural stability training, strength training, etc).

3) each study had to use an inclusion criterion of giving way or frequent sprains, or to have described the target condition as functional ankle instability (FAI), FI or CAI.

Studies using mixed group design (i.e. groups containing subjects with CAI/FI and healthy controls) were excluded from the review. Studies which assessed the additional effect of adjunctive therapies to neuromuscular training such as taping and stochastic resonance [6,10] were included. However for such studies (i.e. studies examining the additional effect of adjunctive therapies), results and effect sizes were acquired for the neuromuscular training groups only. The additional effects of adjunctive interventions were deemed to be beyond the scope of this study.

### Risk of Bias Assessment

Risk of bias in the included studies was assessed by the PI, using the Cochrane collaboration's tool for assessing such risk [11]. This tool was adapted for the objective of this review and consists of 5 domains, with 11 items in total (*see *additional file 2). Each item was rated as 'yes', 'no', or 'unsure'. Studies with 6 or more points on the risk of bias assessment were regarded as having a low risk of bias. This risk of bias tool has previously been utilised by van Rijn et al [12] to investigate the effectiveness of additional supervised exercises compared to conventional treatment alone in patients with acute ankle sprains.

### Data Extraction

The PI extracted relevant data from the included studies. The study characteristics extracted included information on the target population (gender, history of the condition, sample size etc.), presence of concomitant MI, training protocols implemented, outcome measures and significant findings. In cases of uncertainty about the extracted data from the included studies a second reviewer was consulted.

Where feasible the core findings of each article were expressed as effect sizes (ES). If possible, these measures were extracted directly from the article. For articles in which this information was not presented, as was generally the case, effect sizes were calculated using mean values and a pooled standard deviation in accordance with the methods described by Cohen [13]. Effect sizes between 0.2 and 0.49 can be interpreted as weak, 0.5 to 0.79 as medium, and greater than 0.8 as strong [13]. Furthermore, 95% confidence intervals were also calculated.

Outcome measures were grouped into the following categories:

■ Static postural stability

■ Dynamic postural stability

■ Joint position sense

■ Strength measures

■ Muscle onset latencies

■ Joint kinematic data

■ Muscle fatigue values

■ Patient perceived stability

### Data Analysis

The main comparisons of this review were time (i.e. pre and post intervention within the CAI group), and group (i.e. between CAI group and control group) training effects of various neuromuscular training programmes on commonly used sensorimotor outcomes to assess for treatment efficacy in subjects with CAI. Due to the clinical heterogeneity of the trials concerning population, intervention and outcome measures, statistical pooling was not possible. Therefore the data was analysed using a best evidence synthesis as advocated by van Tulder et al [14]. This rating system consists of 4 levels of scientific evidence based on the quality of the included studies:

1) Strong evidence; provided by generally consistent findings in multiple RCTs assessed as having low risk of bias.

2) Moderate evidence; provided by generally consistent findings in one RCT assessed as having low risk of bias, and one or more RCTs assessed as having high risk of bias, or by generally consistent findings in multiple RCTs assessed as having high risk of bias.

3) Limited or conflicting evidence; only one RCT (assessed as having either a low or high risk of bias), or inconsistent findings in multiple RCTs.

4) No available evidence; no published RCTs that have assessed for interventional effect.

## Results

### Literature Search

**Figure 1:**
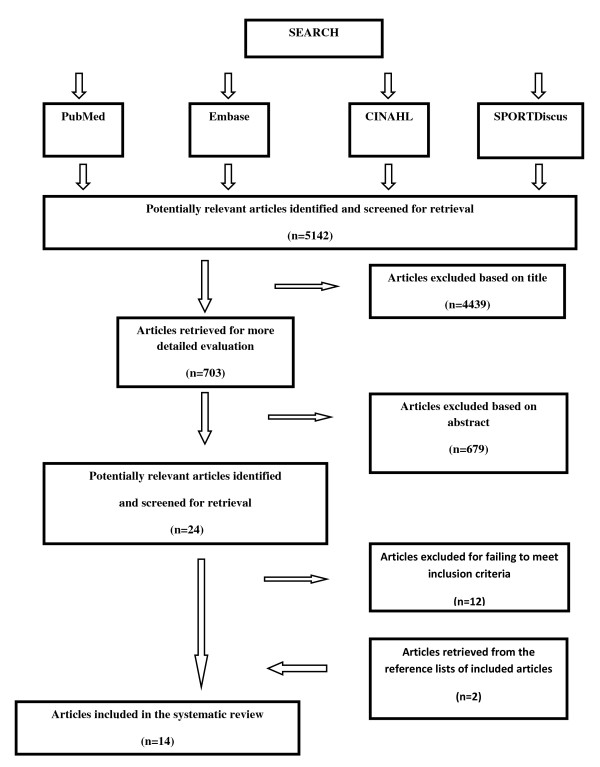
**Flow chart for manuscript review process**.

Our electronic search resulted in 5142 potentially relevant articles. After reviewing titles and abstracts 24 potentially relevant articles remained. Of these, 12 articles met our inclusion criteria after reviewing the full text. A further 2 relevant articles were retrieved after checking the reference lists of included studies. Hence a total of 14 articles were included in this review. The search strategy and results are presented in Figure 1.

### Assessment of Bias

**Figure 2:**
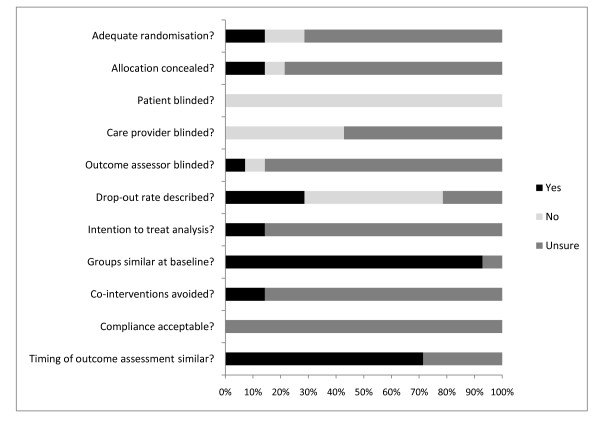
**Results of risk of bias assessment: [frequency (%) of scores per item (yes, no, unsure)]**.

Figure 2 presents the overall assessment of the risk of bias. The assessment of the risk of bias for the individual studies is presented in Table 1. Thirteen of the studies were assessed as having high risk of bias, whilst only one was deemed to be of low risk. The most prevalent shortcomings were found in the items relating to blinding (patient, care provider, outcome assessor), allocation concealment, randomisation, and the acceptability of compliance rates.

**Table 1:** Results of the risk of bias (+ = yes; - = no; ? = unsure)

		1	2	3	4	5	6	7	8	9	10	11
**1**.	**Bernier & Perrin, 1998 **[20]	?	?	-	?	?	+	?	+	?	?	+
**2**.	**Docherty et al, 1998 **[29]	?	?	-	?	?	-	?	+	+	?	?
**3**.	**Rozzi et al, 1999 **[21]	-	-	-	?	?	?	?	+	?	?	?
**4**.	**Matsusaka et al, 2001 **[6]	?	?	-	-	?	?	?	+	?	?	+
**5**.	**Eils & Rosenbaum, 2001 **[22]	?	?	-	?	?	?	?	+	?	?	+
**6**.	**Kaminski et al, 2003 **[32]	?	?	-	?	?	-	?	+	?	?	?
**7**.	**Powers et al, 2004 **[23]	?	?	-	-	+	-	?	?	?	?	+
**8**.	**Clark & Burden, 2005 **[31]	?	?	-	-	?	-	?	+	?	?	+
**9**.	**Kynsburg et al, 2006 **[30]	-	?	-	?	?	-	?	+	?	?	+
**10**.	**Ross et al, 2007 **[10]	?	?	-	?	?	-	?	+	?	?	?
**11**.	**Hale et al, 2007 **[7]	?	?	-	-	?	+	?	+	?	?	+
**12**.	**McKeon et al, 2008 **[8]	+	+	-	?	?	-	?	+	?	?	+
**13**.	**McKeon et al, 2009 **[35]	+	+	-	-	-	+	+	+	+	?	+
**14**.	**Han et al, 2009 **[24]	?	?	-	-	?	+	+	+	?	?	+

1 = Adequate randomisation?; 2 = Allocation concealed?; 3 = Patient blinded?; 4 = Care provider blinded?; 5 = Outcome assessor blinded?; 6 = Drop-out rate described?; 7 = Intention to treat analysis?; 8 = Groups similar at baseline?; 9 = Co-interventions avoided?; 10 = Compliance acceptable?; 11 = Timing of outcome assessment similar?

### Description of Included Studies

Tables 2, 3, 4, 5, 6, 7, 8 and 9 present the characteristics of the included studies. Neuromuscular training in the included studies consisted of a wide variety of proprioceptive and strength training drills. Some studies also implemented protocols combining both interventions. The included studies were considered too heterogeneous to perform a meta-analysis. Therefore, we refrained from pooling and performed a best evidence synthesis. Furthermore, the contrasting nature of the various types of proprioceptive and strength training made it impossible to execute an analysis grouped by type of intervention. For that reason, we described the results of the main comparisons per outcome measure. Tables 10, 11, 12 and 13 present the results of the studies per outcome measure.

**Table 2:** Characteristics of the included studies

Author	Study Population	Presence of MI	Groupings/Intervention	Outcome Measures	Significant Findings	Within Group Effect Sizes	Between Group Effect Sizes
**Bernier & Perrin, 1998 **[20]	48 males & females with FAI	Not specified	Control group (n = 14) - no intervention Sham electrical stimulation group (n = 14) Training group (n = 17) - static & dynamic balance training 3 times a week × 6 weeks	SI & MES in SLS for 4 conditions: stable platform with eyes open and eyes closed, and dynamic platform with eyes open and eyes closed Active and passive JPS data for 7 positions: 15° inversion, 0° degrees neutral, and 10° of eversion, performed at 0° and 25° of plantarflexion. Maximum inversion in 25° plantarflexion was also assessed	Training group showed significant MES improvements over the other 2 groups in AP & ML directions for the stable platform and dynamic platform conditions respectively with eyes closed Significant within training group improvements were also noted in the A/P and M/L directions for both conditions with eyes closed	MES - stable platform, eyes closed: A/P direction: 1.08; 95% CI (10.52-30.48) M/L direction: 1.09; 95% CI (5.28-25.72) MES - dynamic platform, eyes closed: A/P direction: 0.71; 95% CI (68.27-78.73) M/L direction: 0.958; 95% CI (65.25-74.75)	MES - stable platform, eyes closed: A/P direction: 0.99 95% CI (12.13-31.87) M/L direction: 0.92; 95% CI (12.63-33.37) MES - dynamic platform, eyes closed: A/P direction: 0.52; 95% CI (63.9-81.10) M/L direction: 0.55; 95% CI (60.9-78.1)
**Docherty et al, 1998 **[29]	20 healthy college students (10 males, 10 females) with FAI	Not specified	Training group (n = 10) -T-band strengthening 3 times a week × 6 weeks Control group (n = 10) - no intervention	Dorsiflexor and evertor isometric muscle strengths Active JPS data collected at 20° for inversion & plantarflexion, & at 10° for eversion and dorsiflexion	Significant beween group interactions for dorisflexion and eversion strength, and inversion, and plantarflexion JPS Significant improvements in all strength and JPS measures post-test within the training group	Dorsiflexion strength: 2.99; 95% CI (38.51-45.39) Eversion strength: 0.83; 95% CI (34.42-41.48) Inversion JPS: 0.98; 95% CI (2.38-7.22) Eversion JPS: 0.77; 95% CI (1.55-5.15) Dorsiflexion JPS: 0.85; 95% CI (1.56-4.54) Plantarflexion JPS: 1.51; 95% CI (2.51-6.79)	Dorsiflexion strength: 2.93; 95% CI (39.31-45.19) Eversion strength: 1.94; 95% CI (27.77-44.93) Inversion JPS: 1.32; 95% CI (2.92-6.28) Plantarflexion JPS: 1.56; 95% CI (2.06-4.84)

MI = mechanical instability; FAI = functional ankle instability, SI = stability index, MES = modified equilibrium score, JPS = joint position sense, A/P = anterior-posterior, M/L = medial/lateral

**Table 3:** Characteristics of the included studies (continued)

Author	Study Population	Presence of MI	Groupings/Intervention	Outcome Measures	Significant Findings	Within Group Effect Sizes	Between Group Effect Sizes
**Rozzi et al, 1999 **[21]	26 active university students (15 male, 11 female) with and without FAI	Not specified	Training group (n = 13) - unilateral static and dynamic Biodex stability training 3 times a week × 4 weeks Healthy control group (n = 13) - identical training to the FAI group	Biodex generated SIs, recorded for 4 conditions: involved limb at levels 2 and 6, and uninvolved limb at levels 2 and 6 AJFAT scores.	Subjects in both groups demonstrated significant post-training improvements in balance ability at stability levels 2 and 6 Post-training AJFAT scores were significantly better for both groups	SI at level 2: 1.13; 95% CI (2.25-6.31) SI at level 6: 0.73; 95% CI (1.09-2.47) AJFAT Scores: 2.39; 95% CI (19.47-23.41)	No significant between group effect for SI at level 2 or 6 & AJFAT
**Matsusaka et al, 2001 **[6]	22 university students (10 women, 12 men) with unilateral FAI	Present in 73% of subjects, as evidenced by a +ve anterior drawer sign	Tape and exercise group (n = 11, 7 with MI) - ankle disc training 5 times per week × 10 weeks with ankle tape in situ Exercise only group (n = 11, 9 with MI) - identical programme without ankle tape in situ Healthy adult group (n = 21) -tested once to determine normal range of rectangular area values	Postural sway was quantified using rectangular area values taken pretest and at 2,3,4,5,6,8, and 10 weeks of training	In the exercise only group postural sway values improved significantly after 6 weeks and were within the normal range after 8 weeks	Exercise only group: Rectangular area values at 6 weeks: 1.501 12.2-15.5 Rectangular area values at 8 weeks: 1.921 11.6-14	No significant between group effect at 6 & 8 weeks

MI = mechanical instability; FAI = functional ankle instability, +ve = positive; SI = stability index, AJFAT = ankle joint functional assessment tool

**Table 4:** Characteristics of the included studies (continued)

Author	Study Population	Presence of MI	Groupings/Intervention	Outcome Measures	Significant Findings	Within Group Effect Sizes	Between Group Effect Sizes
**Eils & Rosenbaum, 2001 **[22]	30 subjects (18 male, 12 female) with 48 unstable ankles	Not specified	Training group (n = 20, 31 unstable ankles) - multi-station proprioceptive exercises once per week × 6 weeks Control group (n = 10, 17 unstable ankles) - no intervention	Passive JPS was assessed for 10° and 20° of dorsiflexion, and 15° and 30° of plantarflexion Postural Sway in M/L and A/P directions as well as sway distance was assessed in SLS MRTs of TA, PL, and PB following a sudden inversion perturbation Frequency of recurrence at one year follow up	In the exercise group the results showed significant improvements in JPS (except for 10° of DF), postural sway measures, as well as a significant increase in MRTs for PL and PB A significant reduction in frequency of ankle sprains at one year follow up was also noted within the exercise group	JPS at 20° DF: 0.71; 95% CI (1.22-1.68) JPS at 15° PF: 0.90; 95% CI (1.6-2.2) JPS at 30° PF: 0.86; 95% CI (1.87-2.43) Mean Error: 0.98; 95% CI (1.57-1.93) Postural Sway, std dev M/L: 0.26; 95% CI (4.14-4.66) Postural Sway, max sway M/L: 0.48; 95% CI (20.01-22.69) Postural Sway, total sway distance: 0.41; 95% CI (423.66-498.64) MRT of PL: 0.50; 95% CI (60.96-65.44) MRT of PB: 0.54; 95% CI (66.4-70.9)	No significant between group difference was observed
**Kaminski et al, 2003 **[32]	38 (22 men, 16 women) subjects with FAI	Not specified	Strength training group - T-band strengthening of invertors & evertors 3 times per week × 6 weeks Proprioception training group - "T-band kicks" 3 times per week × 6 weeks Coupled strength & proprioception group - both exercise protocols combined Control group no intervention	Isokinetic strength measures of average torque and peak torque eversion to inversion (E/I) ratios, calculated at 30°/sec and 120°/sec	No significant differences in average torque or peak torque E/I ratios for any of the groups	No significant within group effect was observed	No significant between group difference was observed

MI = mechanical instability; FAI = functional ankle instability; JPS = joint position sense; A/P = anterior-posterior; M/L = medial/lateral; SLS = single leg stance; MRT = muscle reaction time; TA = tibialis anterior; PL = peroneus longus; PB = peroneus brevis

**Table 5:** Characteristics of the included studies (continued)

Author	Study Population	Presence of MI	Groupings/Intervention	Outcome Measures	Significant Findings	Within Group Effect Sizes	Between Group Effect Sizes
**Powers et al, 2004 **[23]	38 subjects (22 males, 16 females) with unilateral FAI	Absent on examination	Strength training group - theraband strength training 3 times a week × 6 weeks Proprioceptive training group -proprioceptive training involving "T-band kicks" 3 times a week × 6 weeks Combination training group -performed a combination of both training protocols 3 times a week × 6 weeks Control group - no intervention	Muscle fatigue was determined using the median power frequency (fmed) from an EMG signal for TA and PL COP values for A/P and M/L directions, and the mean overall deviations from COP were obtained	No significant effects of any intervention on measures of muscle fatigue and static balance	No significant within group effect was observed	No significant effect between group effect was observed
**Clarke and Burden, 2005 **[31]	19 male subjects with FAI	Absent on examination	Control group (n = 9) - no intervention Exercise group (n = 10) - wobble board training 3 times a week × 4 weeks	MRTs were measured for TA, and PL in response to sudden inversion AJFAT scores	The exercise group showed a significant decrease in muscle onset latency for both TA and PL, and a significant improvement in AJFAT scores	TA = 1.29 PL = 1.20 Both effect sizes were reported in the paper without presentation of mean ± SD values	Data was presented in graphical format without the reporting of mean ± SD values

MI = mechanical instability; FAI = functional ankle instability; EMG = electromyography, TA = tibialis anterior; PL = peroneus longus; COP = center of pressure; A/P = anterior-posterior; M/L = medial/lateral; MRT = muscle reaction time; AJFAT = ankle joint functional assessment tool; SD = standard deviation

**Table 6:** Characteristics of the included studies (continued)

Author	Study Population	Presence of MI	Groupings/Intervention	Outcome Measures	Significant Findings	Within Group Effect Sizes	Between Group Effect Sizes
**Kynsburg et al, 2006 **[30]	20 subjects (10 males, 10 females):10 with unilateral FAI, 10 healthy matched controls	Not specified	FAI training group (n = 10) -single leg proprioceptive training 3 times per week × 6 weeks Healthy control group (n = 10) - no intervention	Active JPS was measured using the slope-box test for 11 different slope amplitudes in 4 directions (anterior, posterior, lateral, and medial).	Within the training group there was a significant improvement in JPS error in the posterior direction, as well as an overall improvement of the mean absolute estimate error	Posterior JPS: 0.47; 95% CI (1.76-5.0) Cumulative JPS: 0.40; 95% CI (1.99-5.43)	Insufficient data Control group mean ± SD values are not reported in the paper
**Ross et al, 2007 **[10]	30 subjects (16 females, 14 males) with FAI	Majority of subjects had MI (67% with a positive anterior drawer, 76% with talar tilt laxity)	Coordination training group (n = 10) - single leg coordination training 3 times a week × 6 weeks SR coordination training group (n = 10) - identical exercises but received SR stimulation during training Control group (n = 10) - no intervention	COP measures: A/P sway velocity, M/L sway velocity, M/L standard deviation, M/L maximum excursion, and area	The control and coordination group posttest outcomes were not significantly different for any of the measures recorded	No significant within group effect was observed	No significant effect between group effect was observed

MI = mechanical instability; FAI = functional ankle instability; JPS = joint position sense; COP = center of pressure; A/P = anterior-posterior; M/L = medial/lateral

**Table 7:** Characteristics of the included studies (continued)

Author	Study Population	Presence of MI	Groupings/Intervention	Outcome Measures	Significant Findings	Within Group Effect Sizes	Between Group Effect Sizes
**Hale et al, 2007 **[7]	48 subjects (28 females, 20 males), 29 with CAI and 19 healthy controls	Not specified	FAI training group (n = 16) - 4 weeks of training which addressed ROM, strength, neuromuscular control, and functional tasks. Subjects visited the lab on 6 occasions over the 4 weeks, and exercised 5 times per week at home FAI control group (n = 13) - no intervention Healthy control group (n = 19) - no intervention	COP velocity in SLS with eyes open and closed SEBT measures taken in all 8 directions FADI and FADI-Sport scores	Following rehabilitation, the FAI group had significantly greater SEBT reach improvements on the involved limb than the other two groups in the posteromedial, posterolateral, and lateral directions as well as the mean of all 8 reach directions. Similarly, the CAI-rehab group showed showed significant improvements over the CAI-control group, and the healthy group, for FADI and FADI-Sport scores	Pre to post-test scores are presented in the paper for the CAI group as follows (values are presented as % change): P/M: 0.07; 95% CI (0.02-0.12) L: 0.09; 95% CI (0.04-0.08) P/L: 0.12; 95% CI (0.06-0.18) FADI: 7.30; 95% CI (2.47-12.13) FADI Sport: 11.10; 95% CI (6.35-15.86)	Insufficient data was presented for the calculation of between group effect sizes

MI = mechanical instability; CAI = chronic ankle instability; ROM = range of movement; COP = center of pressure; SEBT = Star Excursion Balance Test; FADI = foot and ankle disability index; P/M = posterior-medial; L = lateral; P/L = posterior-lateral

**Table 8:** Characteristics of the included studies (continued)

Author	Study Population	Presence of MI	Groupings/Intervention	Outcome Measures	Significant Findings	Within Group Effect Sizes	Between Group Effect Sizes
**McKeon et al, 2008 **[8]	31 physically active individuals (12 males, 19 females) with a history of FAI	Not specified	CAI balance training group (n = 16) - balance training that emphasised dynamic stabilisation in SLS 3 times per week × 4 weeks CAI control group (n = 15) - no intervention	FADI and FADI-Sport scores COP excursion measures including a 95% confidence ellipse, velocity, range and SD TTB measures including the absolute minimum TTB, mean of TTB minima, and SD of TTB minima in the A/P and M/L directions with eyes open and closed SEBT measures in the A/P, P/M, and P/L directions	The balance training group had significant improvements in the FADI and the FADI-Sport scores, in the magnitude and variability of TTB measures with eyes closed, and in reach distances in the posteromedial and posterolateral directions of the SEBT. Only one of the summary COP-based measures (velocity of COPML, eyes closed) significantly changed after balance training	FADI Scores: 0.98; 95% CI (86.35-92.85) FADI-Sport Scores: 1.25; 95% CI (72.0-82.9) Absolute Min TTB M/L eyes closed: 0.8; 95% CI (0.48-0.56) Mean Min TTB M/L eyes closed: 0.6; 95% CI (1.77-2.23) Mean min TTB A/P eyes closed: 0.41; 95% CI (4.93-6.43) SD Min TTB A/P eyes closed: 0.75; 95% CI (3.05-3.97) Velocity of COP A/P eyes open: 0.07; 95% CI (0.64-0.84) Velocity of COP M/L eyes closed: 0.52; 95% CI (1.85-2.27) SEBT P/M reach: 0.64; 95% CI (0.81-0.93) SEBT P/L reach: 0.67; 95% CI (0.76-0.88)	FADI Scores: 0.68; 95% CI (82.13-92.97) FADI-Sport Scores: 1.63; 95% CI (70.09-81.21) Absolute Min TTB M/L eyes closed: 0.60; 95% CI (0.49-0.57) Mean Min TTB M/L eyes closed: 0.54; 95% CI (1.79-2.25) MeanMinTTB A/P eyes closed: 0.32; 95% CI (4.76-6.09) SD Min TTB A/P eyes closed: 1.18; 95% CI (3.02-3.86) Velocity of COP A/P eyes open: 0.38; 95% CI (0.66-0.8) Velocity of COP M/L eyes closed: 0.42; 95% CI (1.81-2.23) SEBT P/M reach: 1.83; 95% CI (0.82-0.9) SEBT P/L reach: 1.0; 95% CI (0.77-0.88)

MI = mechanical instability; CAI = chronic ankle instability; FADI = foot and ankle disability index; COP = center of pressure; TTB = time-to-boundary; SD = standard deviation; SEBT = Star Excursion Balance Test; A/P = anterior-posterior; M/L = medial/lateral; P/M = posterior-medial; P/L = posterior-lateral; Min = minimum

**Table 9:** Characteristics of the included studies (continued)

Author	Study Population	Presence of MI	Groupings/Intervention	Outcome Measures	Significant Findings	Within Group Effect Sizes	Between Group Effect Sizes
**McKeon et al, 2009 **[35]	31 physically active individuals (12 males, 19 females)	Not specified	CAI balance group (n = 17) - training designed to challenge recovery of single limb balance 3 times per week × 4 weeks CAI control group (n = 15) - no intervention	Kinematic measures of rearfoot inversion/eversion, shank rotation, and the coupling relationship of these two segments throughout the gait cycle were taken whilst walking and running	A significant decrease was noted in the shank/rearfoot coupling variabilty during walking as measured by the deviation phase within the balance training group, and between the balance training group and the control group at post-test	Shank/rearfoot coupling: 0.62; 95% CI (11.71-17.59)	Shank/rearfoot coupling: 0.59; 95% CI (11.42-17.89)
**Han et al, 2009 **[24]	40 subjects (20 males, 20 females)	Not specified	CAI exercise group (n = 10) - resisted "T-band kicks" 3 times per week × 4 weeks CAI control group (n = 10) - no intervention Healthy normals exercise group (n = 10) - exercise programme as per CAI exercise group Healthy normals control group (n = 10) - no intervention	TDT of the COP in SLS at 4 and 8 weeks	Balance training significantly improved in subjects with and without a history of FAI. Furthermore, the exercise programme caused a significant improvement in balance for the FAI exercise group when compared to the FAI control group and the healthy normal group	Insufficient data No mean ± SD data presented for calculation	Insufficient data No mean ± SD data presented for calculation

MI = mechanical instability; CAI = chronic ankle instability; TDT = total distance travelled; COP = center of pressure; SLS = single leg stance; SD = standard deviation

**Table 10:** Results of studies per outcome

OUTCOME	DESCRIPTION	STUDIES	TIME EFFECT	GROUP EFFECT	BEST EVIDENCE SYNTHESIS (TIME)	BEST EVIDENCE SYNTHESIS (GROUP)
**Static Postural Stability**	**S.I. for 8 conditions**					
	Stable platform (E.O) AP	1 HR RCT	NO	NO	LENE	LENE
	Stable platform (E.O) ML	1 HR RCT	NO	NO	LENE	LENE
	Stable platform (E.C) AP	1 HR RCT	NO	NO	LENE	LENE
	Stable platform (E.C) ML	1 HR RCT	NO	NO	LENE	LENE
	Dynamic platform (E.O) AP	1 HR RCT	NO	NO	LENE	LENE
	Dynamic platform (E.O) ML	1 HR RCT	NO	NO	LENE	LENE
	Dynamic platform (E.C) AP	1 HR RCT	NO	NO	LENE	LENE
	Dynamic platform (E.C) ML	1 HR RCT	NO	NO	LENE	LENE
	**MES for 8 conditions**					
	Stable platform (E.O) AP	1 HR RCT	NO	NO	LENE	LENE
	Stable platform (E.O) ML	1 HR RCT	NO	NO	LENE	LENE
	Stable platform (E.C) AP	1 HR RCT	YES	YES	LEOE	LEOE
	Stable platform (E.C) ML	1 HR RCT	YES	NO	LEOE	LENE
	Dynamic platform (E.O) AP	1 HR RCT	NO	NO	LENE	LENE
	Dynamic platform (E.O) ML	1 HR RCT	NO	NO	LENE	LENE
	Dynamic platform (E.C) AP	1 HR RCT	YES	NO	LEOE	LENE
	Dynamic platform (E.C) ML	1 HR RCT	YES	YES	LEOE	LEOE
	**Biodex Generated Stability Indices**					
	Involved limb at level 2	1 HR RCT	YES	YES	LEOE	LEOE
	Involved limb at level 6	1 HR RCT	YES	YES	LEOE	LEOE
	**COP Values**					
	COP Area (E.O)	3 HR RCTS	YES, NO, NO	YES, NO, NO	CE	CE
	COP M/L (E.O)	2 HR RCTS	NO, NO	NO, NO	MENE	MENE
	COP A/P (E.O)	2 HR RCTS	NO, NO	NO, NO	MENE	MENE
	COP Total (E.O)	1 HR RCT	YES	N/A	LEOE	LEOE
	A/P COP vel (E.O)	2 HR RCTS	NO, YES	NO, NO	CE	MENE
	A/P COP vel (E.C)	1 HR RCT	NO	NO	LENE	LENE
	M/L COP vel (E.O)	2 HR RCTS	NO, NO	NO, NO	MENE	MENE
	M/L COP vel (E.C)	1 HR RCT	YES	YES	LEOE	LEOE
	A/P COP sd (E.O)	1 HR RCT	NO	NO	LENE	LENE
	A/P COP sd (E.C)	1 HR RCT	NO	NO	LENE	LENE
	M/L COP sd (E.O)	2 HR RCTS	NO, NO	NO, NO	MENE	MENE
	M/L COP sd (E.C)	1 HR RCT	NO	NO	LENE	LENE
	M/L COP Max (E.O)	1 HR RCT	NO	NO	LENE	LENE
	COP Area (E.C)	1 HR RCT	NO	NO	LENE	LENE
	Range of COP AP (E.O)	1 HR RCT	NO	NO	LENE	LENE
	Range of COP AP (E.C)	1 HR RCT	NO	NO	LENE	LENE
	Range of COP ML (E.O)	1 HR RCT	NO	NO	LENE	LENE
	Range of COP ML (E.C)	1 HR RCT	NO	NO	LENE	LENE
	COP vel (E.O)	1 HR RCT	N/A	NO	NAE	LENE
	COP vel (E.C)	1 HR RCT	N/A	NO	NAE	LENE

E.0. = eyes open

E.C. = eyes closed LEOE = limited evidence of effectiveness

HR RTC = high risk randomised controlled trial

CE = conflicting evidence

LR RTC = low risk randomized controlled trial

MENE = moderate evidence, no effectiveness

LENE = limited evidence, no effectiveness

NAE = no available evidence

S.I. = stability index

**Table 11:** Results of studies per outcome

OUTCOME	DESCRIPTION	STUDIES	TIME EFFECT	GROUP EFFECT	BEST EVIDENCE SYNTHESIS (TIME)	BEST EVIDENCE SYNTHESIS (GROUP)
**Static Postural Stability (cont.)**	**Time to Boundary (TTB) Measures:**					
	Abs. Min TTBML (E.O)	1 HR RCT	NO	NO	LENE	LENE
	Abs. Min TTBML (E.C)	1 HR RCT	YES	YES	LEOE	LENE
	Abs. Min TTBAP (E.O)	1 HR RCT	NO	NO	LENE	LENE
	Abs. Min TTBAP (E.C)	1 HR RCT	NO	NO	LENE	LENE
	Mean Min TTBML (E.O)	1 HR RCT	NO	NO	LENE	LENE
	Mean Min TTBML (E.C)	1 HR RCT	YES	YES	LEOE	LENE
	Mean Min TTBAP (E.O)	1 HR RCT	NO	NO	LENE	LENE
	Mean Min TTBAP (E.C)	1 HR RCT	YES	YES	LEOE	LENE
	SD Min TTBML (E.O)	1 HR RCT	NO	NO	LENE	LENE
	SD Min TTBML (E.C)	1 HR RCT	NO	NO	LENE	LENE
	SD Min TTBAP (E.O)	1 HR RCT	NO	NO	LENE	LENE
	SD Min TTBAP (E.C)	1 HR RCT	YES	YES	LEOE	LENE
	**Total Distance Travelled Measure**					
	Involved limb	1 HR RCT	NO	NO	LENE	LENE
**Dynamic Postural Stability**	**SEBT Measures**					
	Anterior	2 HR RCTS	N/A, NO	NO, NO	LENE	MENE
	Posterior	1 HR RCT	N/A	NO	N/A	LENE
	Lateral	1 HR RCT	N/A	YES	N/A	LEOE
	Medial	1 HR RCT	N/A	NO	N/A	LENE
	Anteromedial	1 HR RCT	N/A	NO	N/A	LENE
	Anterolateral	1 HR RCT	N/A	NO	N/A	LENE
	Posteromedial	2 HR RCTS	N/A, YES	YES, YES	LEOE	MENE
	Posterolateral	2 HR RCTS	N/A, YES	YES, YES	LEOE	MENE
	Mean of all 8 directions	1 HR RCT	N/A	YES	N/A	LEOE

Abs. Min = absolute minimum

Mean Min = mean minimum

SD Min = standard deviation of the minimum

TTBAP = time to boundary anteroposteriorly

TTBML = time to boundary mediolaterally

SEBT = star excursion balance test

HR RCT = high risk randomized controlled trial

LENE = limited evidence, no effectiveness

LEOE = limited evidence of effectiveness

MENE = moderate evidence, no effectiveness

E.0. = eyes open E.C. = eyes closed

**Table 12:** Results of studies per outcome

OUTCOME	DESCRIPTION	STUDIES	TIME EFFECT	GROUP EFFECT	BEST EVIDENCE SYNTHESIS (TIME)	BEST EVIDENCE SYNTHESIS (GROUP)
**Joint Position Sense (JPS)**	**Active JPS (NWB)**					
	15° Inversion	1 HR RCT	NO	NO	LENE	LENE
	20° Inversion	1 HR RCT	YES	YES	LEOE	LEOE
	15° Inversion at 25°plantarflexion	1 HR RCT	NO	NO	LENE	LENE
	Maximal Inversion	1 HR RCT	NO	NO	LENE	LENE
	10° Eversion	2 HR RCTS	NO, YES	NO, NO	CE	MENE
	10° Eversion at 25°plantarflexion	1 HR RCT	NO	NO	LENE	LENE
	0° Neutral	1 HR RCT	NO	NO	LENE	LENE
	0° Neutral at 25°plantarflexion	1 HR RCT	NO	NO	LENE	LENE
	10° Dorsiflexion	1 HR RCT	YES	YES	LEOE	LEOE
	20° Plantarflexion	1 HR RCT	YES	YES	LEOE	LEOE
	**Active JPS (WB)**					
	Anterior	1 HR RCT	NO	N/A	LENE	NAE
	Posterior	1 HR RCT	YES	N/A	LEOE	NAE
	Lateral	1 HR RCT	NO	N/A	LENE	NAE
	Medial	1 HR RCT	NO	N/A	LENE	NAE
	Overall	1 HR RCT	YES	N/A	LEOE	NAE
	**Passive JPS (NWB)**					
	15° Inversion	1 HR RCT	NO	NO	LENE	LENE
	15° Inversion at 25°plantarflexion	1 HR RCT	NO	NO	LENE	LENE
	Maximal Inversion	1 HR RCT	NO	NO	LENE	LENE
	10° Eversion	1 HR RCT	NO	NO	LENE	LENE
	10° Eversion at 25°plantarflexion	1 HR RCT	NO	NO	LENE	LENE
	0° Neutral	1 HR RCT	NO	NO	LENE	LENE
	0° Neutral at 25°plantarflexion	1 HR RCT	NO	NO	LENE	LENE
	10° Dorsiflexion	1 HR RCT	YES	N/A	LEOE	NAE
	20° Dorsiflexion	1 HR RCT	YES	N/A	LEOE	NAE
	15° Plantarflexion	1 HR RCT	YES	N/A	LEOE	NAE
	30° Plantarflexion	1 HR RCT	YES	N/A	LEOE	NAE

NWB = non-weight bearing

WB = weight-bearing

HRRCT = high risk randomised control trial

LENE = limited evidence, no effectiveness

LEOE = limited evidence of effectiveness

CE = conflicting evidence

MENE = moderate evidence, no effectiveness

NAE = No available evidence

**Table 13:** Results of studies per outcome

OUTCOME	DESCRIPTION	STUDIES	TIME EFFECT	GROUP EFFECT	BEST EVIDENCE SYNTHESIS (TIME)	BEST EVIDENCE SYNTHESIS (GROUP)
**Muscle Onset Latencies**	**Muscle Reaction Times**					
	30° Tilt TA	1 HR RCT	NO	N/A	LENE	NAE
	20° Inversion TA	1 HR RCT	YES	N/A	LEOE	NAE
	30° Tilt PL	1 HR RCT	YES	N/A	LEAE	NAE
	20° Inversion PL	1 HR RCT	YES	N/A	LEOE	NAE
	30° Tilt PB	1 HR RCT	YES	N/A	LEAE	NAE
**Strength**	**Isometric Strength**					
	Isometric Dorsiflexion	1 HR RCT	YES	YES	LEOE	LEOE
	Isometric Eversion	1 HR RCT	YES	YES	LEOE	LEOE
	**Isokinetic E/I Ratios**					
	Average Torque at 30°/sec	1 HR RCT	NO	NO	LENE	LENE
	Peak Torque at 30°/sec	1 HR RCT	NO	NO	LENE	LENE
	Average Torque at 120°/sec	1 HR RCT	NO	NO	LENE	LENE
	Peak Torque at 120°/sec	1 HR RCT	NO	NO	LENE	LENE
**Muscle Fatigue**						
	Median Power Frequency TA	1 HR RCT	NO	NO	LENE	LENE
**Joint Kinematics**						
	Rearfoot Position	1 LR RCT	NO	NO	LENE	LENE
	Shank Rotation	1 LR RCT	NO	NO	LENE	LENE
	Shank/Rearfoot Coupling	1 LR RCT	YES	YES	LEOE	LEOE
**Frequency of Injury Recurrence**						
	Incidence at 1 year follow up	1 HR RCT	YES	N/A	LEOE	NAE
**Patient Perceived Functional Stability**						
	AJFAT	2 HR RCTS	YES, YES	YES, N/A	MEOE	LEOE
	FADI	2 HR RCTS	N/A, YES	YES, YES	LEOE	MEOE
	FADI-Sport	2 HR RCTS	N/A, YES	YES, YES	LEOE	MEOE

TA = tibialis anterior

MEOE = moderate evidence of effectiveness

PL = peroneus longus

AJFAT = ankle joint functional assessment tool

PB = peroneus brevis

FADI = foot and ankle disability index

LENE = limited evidence, no effectiveness

HR RCT = high risk randomised controlled trial

LEOE = limited evidence of effectiveness

LR RCT = low risk randomised controlled trial

MENE = moderate evidence, no effectiveness

NAE = no available evidence

LEAE = limited evidence, adverse effect

### Effectiveness of Neuromuscular Training

#### Static Postural Stability

Static postural stability impairments have frequently been associated with CAI [15-17], and have predicted ankle sprain injury in physically active individuals [18,19]. Hence, the assessment of static postural stability in single leg stance (SLS) is one method of determining, the efferent, or muscular response to afferent stimulation.

Nine studies described static postural stability as an outcome measure, all of which had a high risk of bias [6-8,10,20-24]. Static postural stability was measured using a multitude of different measures thereby making comparisons between studies extremely difficult. Bernier and Perrin [20] looked at the effect of 6 weeks of static and dynamic postural stability training on sway index (SI) measures, and modified equilibrium scores (MES). Measures were taken for weight-bearing SLS under both static and dynamic conditions, with and without visual cues. Outcomes were obtained for both the anteroposterior (AP) and mediolateral (ML) directions. Based on this one high risk RCT there is limited evidence for both time and group effect for a number of static and dynamic MES scores post training, namely the stable platform AP, and dynamic platform ML conditions. For two other MES conditions, namely the stable platform ML, and dynamic platform AP conditions, there was limited evidence of time but not group effect following the intervention. This effect was only apparent whilst subjects were tested under the eyes closed condition. No such effect was evident under the eyes open test condition. Based on the same high risk RCT there is limited evidence of neither time nor group effect for neuromuscular training for any of the 8 different SI measurements (i.e. stable and dynamic platform conditions in the AP and ML directions, with and without visual cues), or the 4 other MES conditions (i.e. stable and dynamic platform conditions in the AP and ML directions, with eyes open).

Based on another high risk study [21], which investigated the effect of 6 weeks of theraband strengthening in various planes of talocrural and subtalar joint motion, there is limited evidence of both time and group effect for two Biodex Stability System generated stability indices obtained in SLS.

McKeon et al [8] assessed the effect of 4 weeks of postural stability training drills that emphasised dynamic stabilisation in SLS on a variety of centre of pressure (COP) excursion, and time-to- boundary (TTB) measures obtained in SLS. The COP measures included a 95% confidence ellipse, velocity, range, and standard deviation (SD), and were ascertained for both the AP and ML directions with and without visual cues. The TTB measures included the absolute minimum TTB, mean of TTB minima, and SD of TTB minima, in both AP and ML directions with eyes open and eyes closed. Based on this single high risk RCT there is limited evidence for time and group improvements for COP velocity values in a ML direction under the eyes closed condition post training. There is also limited evidence of both time and group effects for a number of TTB measures including the absolute minimum TTBML, mean minimum TTBML, mean minimum TTBAP, and SD minimum TTBAP, all of which occurred under the eyes closed test condition. There was limited evidence of neither group nor time effect following neuromuscular training for any of the other COP or TTB measures evaluated. Based on another high risk RCT [22], which looked at the effect of 6 weeks of multi-station proprioceptive exercises on COP excursions, there is limited evidence to support a time effect for COP total measures with eyes open following training.

Based on three high risk RCTs [6,8,10], there is conflicting evidence regarding improvements in time and group effect for COP area values assessed in SLS, with eyes closed following neuromuscular training. Matsusaka et al [6], and Ross et al [10] looked at the efficacy of single leg coordination training over 10 and 6 weeks respectively, whilst McKeon et al [8] assesed the efficacy of 4 weeks of balance training that emphasised dynamic stabilisation in SLS. Based solely on the study by Ross et al [10], there is limited evidence of no effectiveness following training for time or group improvements in ML COP Max measures with eyes open. Based on two high risk RCTs [22,23], there is moderate evidence of no effectiveness for strength or proprioceptive training on COP ML and AP measures when assessed with eyes open. Based on two other high risk RCTs [8,10] there is moderate evidence of no effect for both time and group conditions for ML COP velocity, or ML COP SD values when assessed with eyes open. Furthermore based on these two studies there is moderate evidence of no group effect for AP COP velocity measures, and conflicting evidence regarding time effect after training, when assessed with eyes open.

Based on one other high risk RCT [24] there is limited evidence of no effect for both time and group conditions for total distance travelled when assessed with eyes open.

#### Dynamic Postural Stability

Two high risk studies [7,8] described dynamic postural stability as an outcome measure. Both studies utilised the Star Excurion Balance Test (SEBT). Deficits in dynamic balance, as measured by the SEBT, have consistently been demonstrated in those with CAI [25-27].

Hale et al [7] looked at between group differences for all 8 directions of the SEBT, whereas McKeon et al [8] analysed time and group effects in the anterior, posteromedial and posterolateral directions only. Based on these two studies there is moderate evidence of group effect for improvements in reach distance in the posteromedial and posterolateral directions of the SEBT following neuromuscular training. There is moderate evidence of no group effect in the anterior direction. Based solely on the study by McKeon et al [8], there is limited evidence of time effect in the posteromedial and posterolateral directions. Based on the study by Hale et al [7], there is limited evidence of group effect in the lateral direction, and for the mean of all 8 directions of the SEBT. There is limited evidence of no effectiveness, or no available evidence to support time or group effects for all other components of the SEBT.

#### Joint Position Sense

Another proprioceptive measure commonly used to assess for improvements post training in subjects with CAI is joint position sense (JPS). Mechanoreceptors are sensitive to pressure and tension caused by dynamic movement and static positions. Hence if mechanoreceptor function is disrupted as is the case in subjects with CAI this often presents as reduced acuity in sensing joint position thereby leading to increased joint position errors. Konradsen and Magnusson [28] reported that an inversion error greater than 7 degrees would equal a 5 mm drop of the lateral border of the foot, which would lead to a hyper-invered foot position at initial contact therefore increasing the potential for injury.

In total 4 high risk studies looked at JPS. Bernier and Perrin [20], and Docherty et al [29] looked at active JPS in non weight-bearing (NWB) following 6 weeks of balance training, and strength training respectively. Kynsburg et al [30] looked at active JPS in WB using the slope box method of analysis pre and post 6 weeks of proprioceptive training. NWB passive JPS was also analysed in 2 studies [20,21] following 6 weeks of proprioceptive training. Based on one high risk RCT [29] there is limited evidence of both time and group effects for significant improvements in joint acuity for 20 degrees inversion, 10 degrees dorsiflexion, and 20 degrees plantarflexion following neuromuscular training. Based on two studies [20,29] there is conflicting evidence regarding time effect, and moderate evidence of no group effect for improvement in JPS for 10 degrees of eversion. Based on the study by Bernier and Perrin [20] there is limited evidence of neither time nor group effect for active or passive angle reproduction at 15 degrees inversion, 0 degrees of neutral, 10 degrees of eversion, the aforementioned angles repeated at 25 degrees of plantarflexion, or maximal inversion which was defined as minus 5 degrees from each individual's maximum inversion active range. There is limited evidence of time effect in the posterior and combined directions of active WB JPS based on the high risk study by Kynsburg et al [30]. Based on the same study there is limited evidence of no time effect in the anterior, medial and lateral directions. Group effects were not analysed in this study. Based on another high risk study [22] there is limited evidence of time effect improvements in angle reproduction for 10 and 20 degrees of dorsiflexion, as well as 15 and 30 degrees of plantarflexion. Again group effects were not calculated in this study.

#### Muscle Onset Latencies

Electromyography (EMG) has been used in the assessment of neuromuscular control as it allows the timing and degree of muscle activity to be determined during functional tasks. Two high risk studies [22,31] looked at muscle onset latencies in response to a sudden inversion perturbation of the ankle joint. Based on the study by Eils and Rosenbaum [22] which looked at muscle reaction times (MRTs) in response to 30 degrees of sudden inversion perturbation there is limited evidence of a prolonged time effect for the peroneus longus (PL) and peroneus brevis (PB) MRTs following 6 weeeks of proprioceptive training. Whilst this finding was at odds with the reduction in muscle onset latencies that was anticipated, the authors did however report on a more synchronised reaction of the PL and tibialis anterior (TA) in stabilising the ankle joint after sudden perturbation. Based on the same study there is limited evidence of no time effect improvement for TA onset post interevention. The authors failed to describe group effects. Based on the study by Clarke and Burden [31], which recorded MRTs in response to a sudden 20 degree inversion of the ankle via a trapdoor mechanism, there is limited evidence for time and group improvements for both TA and PL reaction times following 4 weeks of wobble board training.

#### Strength

Strength ratios have also been used to detect post training improvements in subjects with CAI. Two high risk studies looked at strength measures. Docherty et al [29] assessed isometric dorisflexor and evertor strengths using a handheld dynamometer after 6 weeks of resisted theraband exercises. Kaminski et al [32] looked at isokinetic eversion/inversion (E/I) strength ratios after theraband strengthening, proprioceptive training incorporating "T-band kicks", and a combination of both protocols. This ratio expresses the viewpoint of the evertors acting concentrically to counteract the violent inversion mechanism in an open kinetic chain, and/or the invertors acting eccentrically to slow the lateral displacement of the tibia in a closed kinetic chain scenario. Based on the study by Docherty et al [29] there is limited evidence of both time and group effects for isometric dosiflexion and eversion strengths following this type of neuromuscular training. Based on the study by Kaminski et al [32] there is limited evidence of neither time nor group effect for average or peak torques calculated at 30 degrees/second and 120 degrees/second for any of the training groups.

#### Muscle Fatigue

It has been show that muscle fatigue can significantly impair postural control [33,34]. Thus, it is plausible that improvements in muscle strength and endurance through training would improve stability. One high risk RCT [23] looked at measures of median power frequency (fmed) from an EMG signal to assess for improvements in measures of muscle fatigue in the TA and PL following either resisted strength training, proprioceptive training, or a combination of both. Based on this study there is limited evidence of neither time nor group effect for improvements in measures of muscle fatigue for any of the training groups.

#### Joint Kinematics

One low risk RCT [35] looked at joint kinematics whilst walking and running on a threadmill. Kinematic measures of rearfoot inversion/eversion, shank rotation, and the coupling relationship between these two segments was analysed throughout the gait cycle whilst walking and running. Based solely on this study there is limited evidence of both time and group improvements for improved shank/rearfoot coupling variability during walking as measured by the deviation phase following 4 weeks of balance training. There is limited evidence of neither time nor group effectiveness for improvement in measures of rearfoot position, or shank rotation during walking or running. Equally there is limited evidence of no effect for time nor group improvements for shank/rearfoot coupling whilst running following balance training.

#### Frequency of Recurrence

Incidence of recurrence at one year follow up was assessed by only one high risk RCT [22]. Based on this study there is limited evidence of time effect following the 6 week neuromuscular intervention. The authors did not report on group effects.

#### Patient Perceived Stability

Four high risk studies looked at patient perceived stability scales as an outcome measure. Two trials [21,31] utilised the Ankle Joint Functional Assessment Tool (AJFAT), to assess for the efficacy of 4 weeks of balance training. Two further studies [7,8] used both the Foot and Ankle Disability Index (FADI), and it's sport's sub-section the FADI-Sport to assess for the effectiveness of 4 weeks of balance training on patient perceived stability. The AJFAT is a 12 part questionnaire with the overall score calculated by totalling the point values from the 12 questions (maximum score = 48). The higher the overall score the greater the perceived functional ability of the involved ankle. The FADI is another questionnaire used to quantify self reported disability in subjects with CAI. The FADI contains 26 items related to activities of daily living, and the FADI-Sport contains 8 items that evaluate perceived disability due to foot and ankle injury in endeavours associated with physical activity and sports participation.

Whilst the validity and reliablity of the AJFAT has yet to be established, the reliability and sensitivity of both components of the FADI have previously been reported in subjects with and without FAI [36]. The study by Clarke and Burden [31] looked at time effect only, whereas that of Hale et al [7] looked at group effects only. Hence based on the studies by Rozzi et al [21] and Clarke and Burden [31] there is moderate evidence of time effect improvement in AJFAT scores post neuromuscular training. Based solely on the study by Rozzi et al [21] there is limited evidence for group effect. Based on the studies by Hale et al [7], and McKeon et al [8] there is moderate evidence of group effect for improvements in both FADI and FADI-Sport scores respectively. Based purely on the study by McKeon et al [8] there is limited evidence of time effect for improvements in both the FADI and FADI-Sport scores.

## Discussion

This review summarised the evidence for the effectiveness of neuromuscular training on a variety of sensorimotor and functional deficits in subjects with CAI. In general, this overview revealed only moderate or limited evidence in favour of neuromuscular training, according to outcome measures of static and dynamic postural stability, active and passive JPS, isometric strength, muscle onset latencies, shank-rearfoot coupling, patient perceived stability, and frequency of recurrence. However, for none of the outcome measures strong evidence in favour of neuromuscular training was found.

The aforementioned evidence is based on a limited number of studies (n = 14), with a maximum of eight studies per outcome measure. In these studies neuromuscular training was defined as either proprioceptive drills, strength training, or a combination of both. However, the specific mechanisms of training were quite varible in terms of the mode, frequency, and the duration of the training period. Training protocols varied from 1 session per week for 6 weeks [22], to 5 times per week for 10 weeks [6]. In addition, heterogeneity among the studies was observed concering the study populations in terms of the presence or absence of concommitant MI, and outcome assessment. Furthermore, all but one of the studies included in the review were assessed as having a high risk of bias. Therefore, we refrained from statistical pooling of the results of the individual studies, and instead conducted a best evidence synthesis.

The assesment of risk of bias resulted in almost 93% of the studies identified as having high risk. The threshold to differentiate between low and high risk of bias studies was based on the methodological study of van Tulder et al [14] in which they assessed the validity of the Cochrane Collaboration's tool for assessing the risk of bias in trials with back-pain interventions. In this study a threshold of 50% or less was associated with bias, therefore similar to van Rijn et al [12] it was decided that studies with 6 or more points were regarded as high risk studies. Critical items in the risk of bias assessment were items on randomisation (item 1), allocation concealment (item 2), and blinding (items 3,4, and 5).

None of the studies scored positively on patient or care provider blinding, which is devoted to the fact that the setting of physical therapy often does not lend itself to the blinding of patients or care givers. All of the studies scored "unclear"on the item concerning compliance, and in 86% of the studies it was unclear whether or not co-interventions were avoided. Hence, these studies are more susceptible to selection bias, and as a consequence, the generalisability of the results in this review is adversely effected.

There are a number of plausible explanations to account for the variability in findings among certain studies, and the failure of others to produce statistically significant results. In the studies pertaining to static joint stability [6-8,10,20-24] measures taken in the absence of visual cues tended to produce more meaningful results than those where visual input was retained. Vision is an extremely important sense for the control of balance. It is believed that even when somatosensory input is disrupted due to injury, visual information can provide an adequate amount of feedback to compensate for deficits in the central pathways or the vestibular system [37,38]. Hence, it was perhaps unsurprising that when this compensatory mechanism is removed through closing the eyes, deficits in the sensorimotor system become more apparent. This may be an important consideration for researchers to bear in mind when selecting outcome measures in the future.

Another possible reason for the inconsistent findings among studies is the lack of sensitivity of the measures chosen to detect post training improvements. Many of the studies in the review used traditional COP excursion values to assess for interventional efficacy [6-8,10,22-24]. Unfortunately, these measures have been shown not to be particularly sensitive in detecting CAI related postural control deficits, when compared to TTB measures [17]. TTB measures have also been shown to be more sensitive than traditional COP excursion (COPE) measures in detecting post training improvement in subjects with FAI [8]. These findings may go some way towards explaining why COPE measures have failed to show significant post-training improvements in a number of the studies reviewed. In many of the other studies particularly those relating to strength and JPS [20,22,29,30,32], failure to reveal significant post training effects may be best understood from a mode specificity standpoint, whereby the disparity between training protocols and the outcomes used to assess for efficacy appears to be too great. Researchers examining the area of CAI need to recognise that when subjects are trained using a specific protocol, outcomes that closely resemble the intervention are best suited to assess for treatment effect. Relating to the studies looking at muscle onset latencies [22,31], differences in outcome can be accounted for to some degree due to the different algorithms used to calculate muscle onset latencies. Greater standardisation of testing protocols is required in order for meaningful comparisons to be made.

Furthermore, the majority of studies included in the review examined the efficacy of a specific treatment strategy such as balance training or strength training in isolation. Due to the multi-faceted nature of CAI which cannot be adequately explained through the dichotomy of MI and FI [5], a more comprehensive treatment approach combining strengthening, proprioceptive training, and functional retraining may be more effective in improving lower extremity function and preventing recurrent injury. Addressing local arthrokinematic impairments may also help elicit greater improvements for various outcomes. Following on from this, it may then be beneficial to develop a treatment or impairment based classification system that addresses the multi-factorial nature of the condition. Classification of individuals with CAI into different groups based on impairments or treatment response may lead to more efficient conservative management in the future.

Only one of the studies reviewed [22], looked at recurrence rates at one year follow-up. Hence there is certainly a need for more studies to examine interventional efficacy in the longer term. It is of paramount importance to know if immediate post-training improvements are maintained, and whether or not these improvements carry over to a long-term reduction in symptoms and prevention of injury recurrence. Further research is necessary before any meaningful conclusions can be drawn regarding the efficacy for neuromuscular training leading to improvements in joint kinematics and muscle fatigue. The findings to date relating to patient perceived functional stability look promising, though further reseach will be required to corroborate these preliminary results.

Although deemed to be outside the scope of this review a number of authors have advocated the use of adjuctive therapies such as taping and stochastic resonance stimulation combined with neuromuscular training. Preliminary findings indicate earlier and superior results than training alone [6,10]. Such additional interventions certainly warrant further investigation. Therapies providing a greater treatment effect than neuromuscular training alone may well have implications for improved function, a reduction in injury recurrence, and reduced treatment costs.

## Conclusion

In conclusion, this review showed moderate or limited evidence of effectiveness in favour of neuromuscular training, according to the outcome measures of static and dynamic postural stability, active and passive JPS, isometric strength, muscle onset latencies, shank-rearfoot coupling and injury recurrence rates. For none of the outcome measures strong evidence of effectiveness was found. However, only a small number of studies [14] were eligible for inclusion in the review. Most studies were assessed as having a high risk of bias, and most studies were lacking power. Therefore we recommend conducting further high-quality RCTs with sufficient power to assess for the effectiveness of neuromuscular training in subjects with CAI. Such studies should also consider the importance of mode specificity of training, and the implementation of outcome measures with adequate sensitivity to detect interventional effect

## Competing interests

The authors declare that they have no competing interests.

## Authors' contributions

JOD and ED conceived and performed the study and drafted the manuscript. All authors read and approved the final manuscript.

## Supplementary Material

Additional file 1 **Search terms**. Search terms used for the identification of studies. Click here for file

Additional file 2 **Source of risk bias**. Items used for the assessment of risk bias. Click here for file
