# Supplementary material for: Neuromuscular training to enhance sensorimotor and functional deficits in subjects with chronic ankle instability: A systematic review and best evidence synthesis
Source: Sports Med Arthrosc Rehabil Ther Technol. 2011 Sep 22;3:19. doi: 10.1186/1758-2555-3-19 (PMC3189141; doi:10.1186/1758-2555-3-19)
Supplement: Additional file 1 — Search terms. Search terms used for the identification of studies. [file 1758-2555-3-19-S1.DOCX]

**Additional file 1:** Search terms

1. “ankle injury”
2. “ankle joint”
3. “ligaments”
4. OR/1-3
5. “ankle” in title
6. “sprain” or “strain” or “rupture”
7. “instability” OR “unstable”
8. “repeated” OR “recurrent” OR “multiple”
9. “functional” OR “functionally” OR “chronic” OR “chronically”
10. OR/6-9
11. AND/5, 10
12. AND/4,11
