# Supplementary material for: Neuromuscular training to enhance sensorimotor and functional deficits in subjects with chronic ankle instability: A systematic review and best evidence synthesis
Source: Sports Med Arthrosc Rehabil Ther Technol. 2011 Sep 22;3:19. doi: 10.1186/1758-2555-3-19 (PMC3189141; doi:10.1186/1758-2555-3-19)
Supplement: Additional file 2 — Source of risk bias. Items used for the assessment of risk bias. [file 1758-2555-3-19-S2.DOCX]

**Additional file 2:** Sources of risk bias

**Item Judgement**

**A) Sequence generation**

1. Was the method of randomization adequate? Yes / No / Unsure

**B) Allocation concealment**

2. Was the treatment allocation concealed? Yes / No / Unsure

**C) Blinding of participants, personnel and outcome**

*Was knowledge of the allocated interventions adequately prevented during the study?*

3. Was the patient blinded to the intervention? Yes / No / Unsure

4. Was the care provider blinded to the intervention? Yes / No / Unsure

5. Was the outcome assessor blinded to the intervention? Yes / No / Unsure

**D) Incomplete outcome data**

*Were incomplete outcome data adequately addressed?*

6. Was the drop-out rate described and acceptable? Yes / No / Unsure

7. Were all randomized participants analysed in the group to

which they were allocated? Yes / No / Unsure

**E) Other sources of potential bias**

8. Were the groups similar at baseline regarding the most

important prognostic indicators? Yes / No / Unsure

9. Were co-interventions avoided or similar? Yes / No / Unsure

10. Was the compliance acceptable in all groups? Yes / No / Unsure

11. Was the timing of the outcome assessment similar in all

groups? Yes / No / Unsure
